# Advancing Equity in Left Atrial Appendage Occlusion Outcomes for Hispanic/Latino Patients

**DOI:** 10.1002/clc.70176

**Published:** 2025-07-10

**Authors:** Syed Muhammad Rayyan, Bakhtiyar Ameer, Mueed Iqbal, Muhammad Abdul Haseeb Khan, Farmanullah Khan

**Affiliations:** ^1^ Ayub Medical College Abbottabad Pakistan

To the Editor,

We read with great interest the article published by Fleurestil and his colleagues. Fleurestil and colleagues have provided valuable insights into in‐hospital outcomes after left atrial appendage occlusion (LAAO) among Hispanic/Latino patients using the National Inpatient Sample (NIS). Yet, in striving for equitable stroke prevention, several further considerations merit attention [1].

While the authors note absence of echocardiographic and catheterization details, they do not address variability in device selection (e.g., Watchman vs. Amplatzer), catheter access routes, or operator experience. Each device has distinct learning curves and complication profiles, and proficiency varies markedly across centers. Future studies should partner with the NCDR LAAO Registry—which captures device type, sheath size, fluoroscopy time, and operator volume—to determine whether specific techniques or low‐volume operators disproportionately contribute to the elevated infectious and vascular complications seen in Hispanic/Latino groups [2].

By design, NIS only captures index hospitalization events. Yet device‐related thrombus, late pericardial effusions, and stroke recurrence often manifest weeks to months later. Incorporating linkage to claims data (e.g., Medicare Part A/B) or designing a prospective, multicenter registry with mandatory 12‐month follow‐up would illuminate whether early in‐hospital disparities translate into divergent long‐term safety and efficacy outcomes.

Though Fleurestil et al. briefly cite insurance status and language barriers, they do not quantify health literacy, immigration status, or neighborhood deprivation. Embedding validated instruments—such as the Rapid Estimate of Adult Literacy in Medicine (REALM) [3] or Area Deprivation Index [4]—into future LAAO registries would allow risk adjustment for social determinants and guide culturally tailored peri‐procedural education.

Administrative coding cannot verify periprocedural anticoagulation regimens or post‐implant adherence. Given that suboptimal anticoagulant use may drive both bleeding and thrombotic events, prospective studies should incorporate pharmacy fill data and wearable adherence monitors. Moreover, qualitative interviews could uncover patient‐level barriers to compliance, enabling development of targeted interventions (e.g., bilingual mobile reminders).

With only 6814 Hispanic/Latino cases (4.9%), the study risks type II error for less frequent outcomes and cannot explore heterogeneity within the Hispanic/Latino umbrella (e.g., Caribbean vs. Central American ancestry). Pooling data across international centers, or applying Bayesian hierarchical models, would enhance power and allow disaggregation by cultural background, socioeconomic bracket, and comorbidity clusters.

Clinical endpoints—mortality, bleeding, vascular complications—while critical, overlook quality‐of‐life and patient satisfaction. Incorporating standardized patient‐reported outcome measures (PROMs), such as the Atrial Fibrillation Effect on Quality‐of‐Life (AFEQT) questionnaire [5], would offer a holistic view of how LAAO impacts recovery, symptom burden, and return to daily activities across ethnic groups.

To advance equitable care, we urge the LAAO community to move beyond administrative datasets. Leveraging detailed procedural registries (e.g., NCDR) [2], integrating longitudinal follow‐up, embedding socioeconomic and adherence metrics, employing robust statistical methods, and capturing patient‐centered outcomes will collectively clarify drivers of observed disparities. Only through such comprehensive, multidisciplinary efforts can we tailor LAAO strategies that truly serve the diverse Hispanic/Latino population at risk for atrial fibrillation–related strokes.

Respectfully submitted.

## Ethics Statement

The authors have nothing to report.

## Consent

The authors have nothing to report.

## Conflicts of Interest

The authors declare no conflicts of interest.

## Data Availability

The authors have nothing to report.

## References

[1] M. Fleurestil , A. Mohan , I. Ergui , et al., “Outcomes of Left Atrial Appendage Occlusion in Hispanic/Latino Patients: Insights From the National Inpatient Sample,” Clinical Cardiology 48, no. 4 (2025): 321–329. 10.1002/clc.70152 PMC12076124 40365821

[2] D. R. Lakkireddy and M. K. Turagam , “NCDR Left Atrial Appendage Occlusion Registry,” Journal of the American College of Cardiology 75, no. 13 (April 2020): 1519–1522, 10.1016/j.jacc.2020.01.007. 32238317

[3] T. C. Davis , S. W. Long , R. H. Jackson , et al., “Rapid Estimate of Adult Literacy in Medicine: A Shortened Screening Instrument,” Family Medicine 25, no. 6 (June 1993): 391–395. 8349060

[4] R. Powell , A. Sheehy , and A. J. H. Kind , “The Area Deprivation Index Is the Most Scientifically Validated Social Exposome Tool Available for Policies Advancing Health Equity,” Health Aff Forefront (July 2023), 10.1377/forefront.20230714.676093.

[5] J. Spertus , P. Dorian , R. Bubien , et al., “Development and Validation of the Atrial Fibrillation Effect on QualiTy‐of‐Life (AFEQT) Questionnaire in Patients With Atrial Fibrillation,” Circulation: Arrhythmia and Electrophysiology 4, no. 1 (February 2011): 15–25, 10.1161/CIRCEP.110.958033. 21160035

